# Efficacy and safety of polymer-free amphilimus-eluting stent in patients with and without diabetes mellitus: A prospective, multicenter observational study

**DOI:** 10.1371/journal.pone.0336017

**Published:** 2025-11-10

**Authors:** Subin Lim, Ju Hyeon Kim, Soon Jun Hong, Jung-Joon Cha, Hyung Joon Joo, Jae Hyoung Park, Cheol Woong Yu, Do-Sun Lim, Jae-Youn Moon, Soon Yong Suh, Jin Man Cho

**Affiliations:** 1 Department of Cardiology, Cardiovascular Center, Ewha Womans University Seoul Hospital, Seoul, Republic of Korea; 2 Department of Cardiology, Cardiovascular Center, Korea University Anam Hospital, Korea University College of Medicine, Seoul, Republic of Korea; 3 Department of Cardiology, Cardiovascular Center, Asan Medical Center, Seoul, Republic of Korea; 4 Department of Cardiology, CHA Bundang Medical Center, CHA University, Gyeonggi-do, Republic of Korea; 5 Department of Cardiology, Gachon University Gil Medical Center, Incheon, Republic of Korea; 6 Cardiovascular Center, Kyunghee University Hospital at Gangdong, Seoul, Republic of Korea; IPSurface Canada Inc, CANADA

## Abstract

**Background:**

Patients with diabetes mellitus (DM) undergoing percutaneous coronary intervention face higher risks of restenosis and adverse cardiovascular outcomes compared to those without DM. This study compared the real-world safety and effectiveness of the Cre8/Cre8 EVO stents in patients with and without diabetes.

**Methods:**

We performed an investigator-initiated, prospective, single-arm observational trial at 28 sites in South Korea. The primary endpoint was a composite of cardiac death, target vessel-related myocardial infarction (MI), and any clinically driven repeat revascularization at 12 months. All-cause mortality was a key secondary endpoint. The adjusted outcomes of DM and non-DM groups were compared using 1:1 propensity score (PS) matching.

**Results:**

A total of 2,043 patients (66.0 ± 11.5 years of age; 76.2% male) were analyzed. Diabetic patients (n = 773; HbA1c 7.3 ± 1.4%) were more likely to be older, female, and have hypertension, dyslipidemia, or chronic kidney disease. Among these, 20.2% (156 patients) were using insulin. There were 54 cases of primary endpoint, 22 (cumulative incidence, 3.4%) in the DM group and 32 (3.0%) in the non-DM group (p = 0.61). The DM group exhibited a higher all-cause mortality rate compared to the non-DM group (2.1% vs. 1.3%; p = 0.19). The adjusted risk of 1-year primary endpoint was similar between the DM and non-DM groups (hazard ratio, 1.20; 95% confidence interval, 0.63–2.30), with comparable safety profiles.

**Conclusion:**

In this real-world study, the DM group treated with amphilimus-eluting stents demonstrated sufficient safety and effectiveness at 12 months, with a similar occurrence of cardiovascular events compared to the non-DM group.

## Introduction

Patients with diabetes mellitus (DM) undergoing percutaneous coronary intervention (PCI) are at a significantly higher risk of restenosis [1–3] and adverse cardiovascular outcomes [4–7] compared to those without DM. Despite advancements in drug-eluting stent (DES) technology, cardiovascular outcomes for patients with DM have not significantly improved over the past decade. [8,9] Current evidence suggests minimal differences in outcomes between most contemporary DES in patients with DM [10,11], highlighting the ongoing need for specialized stent designs tailored to this growing patient population.

The polymer-free amphilimus-eluting stents (PF-AES) have a novel design with a thin-strut (80 𝝻m) cobalt-chromium alloy coated with a bio-inducer surface to promote reendothelialization and abluminal reservoirs containing sirolimus and fatty acids (amphilimus formulation) that are released over a 90-day period. [12] Several small randomized and non-randomized studies have shown that PF-AES provides clinical benefits in patients with DM [13–17], likely because of reduced polymer-related inflammation and low thrombogenicity from the thin profile. [18,19] In a recent randomized trial that included 1,175 patients with DM from 23 sites in Spain, the PF-AES were found to be noninferior to contemporary polymer-based zotarolimus-eluting stents in terms of target-lesion failure composite outcomes. [20]

To further explore these findings, we conducted a prospective registry study to evaluate the clinical safety and effectiveness of the PF-AES in an unselected patient population, reflecting real-world practice. The Cre8 EVO is designed to enhance the homogeneous distribution of the antiproliferative drug within the vessel wall, while increasing flexibility and deliverability. This study places particular emphasis on the clinical applicability of Cre8/Cre8 EVO stents in patients with DM and aims to compare the clinical outcomes between diabetic and non-diabetic patients in real-world settings.

## Methods

### Study design and population

This study (ClinicalTrials.gov identifier: NCT05759676) is a prospective, single-arm, observational, multicenter, investigator-initiated trial to enroll 2,000 patients from 28 sites in South Korea between June 29, 2020, and December 31, 2023. The study population consists of consecutive patients who underwent PCI with the implantation of a Cre8™ or Cre8™ EVO stent (CID Spa, Italy – part of Alvimedica, Turkey) for coronary artery disease. Patients aged 19 years or older who consented to the research protocol, submitted written informed consent, and participated in clinical follow-up visits were eligible. Exclusion criteria included the following: known hypersensitivity or contraindications to heparin, aspirin, clopidogrel, amphilimus, cobalt chrome, stainless steel, nickel, 316L metal, and contrast media (if anaphylaxis was known), pregnancy or plans to become pregnant during the study period, planned surgery requiring the cessation of antiplatelet therapy within 12 months, life expectancy of less than 1 year, participation in other medical device randomization studies, or PCI with a stent other than PF-AES at the time of registration. All patients provided written informed consent prior to participation, and the study was approved by the institutional review board of Korea University Anam Hospital (2023AN0041). The trial was carried out in compliance with the Declaration of Helsinki and Good Clinical Practice guidelines.

### Study procedures and measurement

The PCI procedure was performed in accordance with the latest practice recommendations. There were no limitations on the quantity, size, or dimensions of the PF-AES used. The operator had the discretion to utilize a range of drugs, equipment, and procedures, including glycoprotein IIb/IIIa inhibitors, heparin, thrombectomy devices, intravascular ultrasound (IVUS), optical coherence tomography (OCT), and pressure wires. Plain balloon angioplasty, with or without the use of a drug-coated balloon, was allowed in combination with Cre8™/Cre8™ EVO implantation for patients undergoing multivessel or bifurcation PCI. Lesion success was determined by the presence of a residual stenosis of 20% or less and a final Thrombolysis in Myocardial Infarction flow grade of 3, as visually assessed. The attending physicians were advised to evaluate the choice, amount, and duration of dual antiplatelet therapy (DAPT) using aspirin and a P2Y12 inhibitor (clopidogrel, prasugrel, or ticagrelor) in accordance with the current guidelines. [21–24]

Multivessel disease was defined as luminal stenosis of at least 70% in at least two major coronary arteries or in one coronary artery in addition to a 50% or greater stenosis of the left main trunk. Severe coronary calcification was defined as radio-opacities noted without cardiac motion before contrast injection, seen as densities on either side of the arterial wall (tram-track or circular opacities, visible in multiple projections). [25,26]

### Study outcomes

The primary endpoint was major adverse cardiovascular events (MACE), which included cardiac death, target vessel-related myocardial infarction (TVMI), and any clinically driven repeat revascularization at 12 months. The major secondary endpoints included all-cause death and individual components of the primary composite outcome. Clinically driven repeat revascularization included both target lesion revascularization (TLR) and target vessel revascularization (TVR). TLR was defined as a repeat percutaneous intervention of the target lesion or bypass surgery of the target vessel performed for restenosis or other complication of the target lesion. TVR was defined as any repeat percutaneous intervention or surgical bypass of any segment of the target vessel including the target lesion. Target lesion failure (TLF) was defined as a composite of cardiac death, TVMI and TLR. An independent clinical event committee adjudicated all events, and data quality was independently monitored. Unless an incontrovertible non-cardiovascular cause was identified, all deaths were classified as cardiac deaths. MI was defined as elevated cardiac biomarker levels with concomitant ischemic symptoms or signs according to noninvasive (electrocardiography or imaging results) and invasive (coronary angiography) examinations. Periprocedural MI was not included in the definition of MI. Any repeat revascularization can be performed using either PCI or coronary artery bypass graft surgery on either the target or non-target vessels.

Clinical follow-up visits were scheduled for 1, 6, and 12 months after the index PCI, and were conducted either in-office or via telephone as needed. Throughout the follow-up period, it was highly recommended to apply guideline-directed medical therapy and effectively manage risk factors for intensive secondary prevention in accordance with the latest clinical guidelines. At each visit, a thorough collection of all relevant data on cardiovascular drugs and clinical events was prospectively collected in a systematic manner. Survival status was verified via the Korean National Health Insurance Service database.

### Statistical analysis

Continuous variables are reported as means and standard deviations, while categorical variables are expressed as counts and percentages. The parametric unpaired *t*-test or non-parametric Mann–Whitney *U* test was employed to conduct group comparisons for continuous variables, while the χ2 or Fisher’s exact test was conducted for categorical variables. The log-rank test was employed to evaluate intergroup comparisons, and cumulative incidence rates were determined using Kaplan–Meier estimates. Patients were censored at the time of the event or the date of their most recent follow-up. The hazard ratios (HRs) and 95% confidence intervals were calculated using a multivariable Cox proportional hazard regression model to investigate the impact of various covariates on time-to-event outcomes. To account for the differences in baseline characteristics, we conducted a propensity score (PS) matching analysis to compare outcomes between patients with and without DM. The propensity score model was matched for baseline clinical characteristics to adjust for the worse profiles of patients with diabetes. The variables included in the propensity score model were age, sex, body mass index, hypertension, dyslipidemia, smoking, family history of premature coronary artery disease, chronic kidney disease (CKD), end-stage renal disease on dialysis, prior PCI, prior MI, and index presentation as acute MI. Procedural factors, which were determined during or after the procedure, were not included in the matching process. We applied the nearest-neighbor matching method with a 1:1 matching ratio and a caliper width equal to 0.2 of the standard deviation of the logit PS. A standardized mean difference of less than 0.1 indicated a negligible difference. Statistical analyses were conducted using R version 4.1.2 (R Foundation for Statistical Computing, Vienna, Austria), with a p-value of less than 0.05 being considered statistically significant.

## Results

### Demographic and Clinical Characteristics

The study enrolled 2,043 patients, with 773 patients in the DM group and 1,270 in the non-DM group before PS matching. (**Table 1**) The mean age of patients in the DM group (HbA1c 7.3 ± 1.4%) was slightly higher than in the non-DM group (*p* < 0.01). Among patients with DM, 20.2% (156 patients) were using insulin. The prevalence of hypertension and dyslipidemia was significantly higher in the DM group. CKD and end-stage renal disease on dialysis were also more common in the DM group (11.5% vs. 3.2%, *p* < 0.01; and 5.2% vs. 1.1%, *p* < 0.01, respectively).

**Table 1:** Demographic and clinical characteristics before and after propensity score matching.

	Unmatched cohort			Propensity score-matched cohort			
	DM (n = 773)	Non-DM (n = 1270)	*P* value	DM (n = 735)	Non-DM (n = 735)	*P* value	SMD
**Demographics**							
Age (years)	67.5 ± 11.2	65.1 ± 11.6	<0.01	67.5 ± 11.3	66.3 ± 11.3	0.05	−0.0985
Male, n (%)	564 (73.0%)	993 (78.2%)	<0.01	540 (73.5%)	561 (76.3%)	0.23	0.0692
Body mass index, kg/m^2^	24.8 ± 2.8	24.8 ± 2.5	0.73	24.8 ± 2.8	24.9 ± 2.7	0.58	0.0307
**Risk factors,** n (%)							
Hypertension	593 (76.7%)	697 (54.9%)	<0.01	556 (75.6%)	521 (70.9%)	0.05	−0.0957
Dyslipidemia	395 (51.1%)	497 (39.1%)	<0.01	369 (50.2%)	325 (44.2%)	0.03	−0.1227
Smoking	179 (23.2%)	329 (25.9%)	0.18	172 (23.4%)	173 (23.5%)	1.00	0.0031
Family history of CAD	54 (7.0%)	68 (5.4%)	0.16	50 (6.8%)	36 (4.9%)	0.15	−0.0846
**Co-morbidities,** n (%)							
Peripheral artery disease	7 (0.9%)	8 (0.6%)	0.66	6 (0.8%)	4 (0.5%)	0.75	NA
Chronic kidney disease	89 (11.5%)	41 (3.2%)	<0.01	51 (6.9%)	39 (5.3%)	0.23	−0.0924
ESRD on dialysis	40 (5.2%)	14 (1.1%)	<0.01	18 (2.4%)	14 (1.9%)	0.59	−0.0521
Previous PCI	173 (22.4%)	203 (16.0%)	<0.01	157 (21.4%)	127 (17.3%)	0.06	−0.1114
Previous CABG	16 (2.1%)	18 (1.4%)	0.35	14 (1.9%)	11 (1.5%)	0.69	NA
Previous MI	76 (9.8%)	106 (8.3%)	0.29	73 (9.9%)	69 (9.4%)	0.79	−0.0197
Previous stroke	57 (7.4%)	68 (5.4%)	0.08	51 (6.9%)	48 (6.5%)	0.84	NA
Previous ICH	9 (1.2%)	16 (1.3%)	1.00	9 (1.2%)	5 (0.7%)	0.42	NA
**Index presentation as acute MI,** n (%)	218 (28.2%)	440 (34.6%)	<0.01	208 (28.3%)	225 (30.6%)	0.36	0.0486

Values are presented as numbers (percentages) or means ± standard deviation. NA, not applicable due to exclusion from propensity-score matching; SMD, standardized mean difference

CABG = coronary artery bypass graft; CAD = coronary artery disease; DM = diabetes mellitus; ESRD = end-stage renal disease; ICH = intracranial hemorrhage; MI = myocardial infarction.

### Angiographic and Procedural Characteristics

In the unmatched cohort, the DM group had a higher prevalence of multivessel disease (16.8% vs. 14.6%), and severely calcified lesions (25.7% vs. 15.7%) compared to the non-DM group. (**Table 2**) Additionally, patients with DM had a higher prevalence of ACC/AHA lesion type B2/C (76.3% vs. 72.7%) and in-stent restenosis lesions (7.5% vs. 4.2%). The use of intracoronary imaging was lower in the DM group (34.4% vs. 39.8%; *p* = 0.02). The mean stent diameter was slightly smaller in DM patients (3.08 mm vs. 3.14 mm), and the total stent length was also slightly longer in DM patients (34.8 mm vs. 32.9 mm). The overall incidence of procedural complications, including slow flow or no-reflow, dissection, side branch occlusion, distal embolization, acute thrombosis, stent migration, and coronary perforation, was low and comparable between the DM and non-DM groups.

**Table 2:** Angiographic and procedural characteristics before and after propensity score matching.

	Unmatched cohort			Propensity score-matched cohort			
	DM (n = 773)	Non-DM (n = 1270)	*P* value	DM (n = 735)	Non-DM (n = 735)	*P* value	SMD
Indication for index procedure			0.05			0.76	
Silent ischemia	37 (4.8%)	48 (3.8%)		35 (4.8%)	33 (4.5%)		
Stable angina	256 (33.1%)	382 (30.1%)		243 (33.1%)	225 (30.6%)		
Unstable angina	262 (33.9%)	400 (31.5%)		249 (33.9%)	252 (34.3%)		
Non-ST-segment elevation MI	115 (14.9%)	232 (18.3%)		106 (14.4%)	122 (16.6%)		
ST-segment elevation MI	103 (13.3%)	208 (16.4%)		102 (13.9%)	103 (14.0%)		
Cardiogenic shock	15 (1.9%)	21 (1.7%)	0.76	15 (2.0%)	12 (1.6%)	0.70	
Elective PCI	567 (73.4%)	915 (72.0%)	0.56	536 (72.9%)	543 (73.9%)	0.72	
Trans-radial PCI	571 (73.9%)	971 (76.5%)	0.21	552 (75.1%)	560 (76.2%)	0.67	
Preload with P2Y12 inhibitor	495 (64.0%)	846 (66.6%)	0.25	466 (63.4%)	470 (63.9%)	0.87	
IIb/IIIa inhibitor	13 (1.7%)	23 (1.8%)	0.97	13 (1.8%)	14 (1.9%)	1.00	
Number of diseased vessels			<0.01			0.02	
1	356 (46.1%)	663 (52.2%)		345 (46.9%)	375 (51.0%)		
2	222 (28.7%)	376 (29.6%)		211 (28.7%)	225 (30.6%)		
3	195 (25.2%)	231 (18.2%)		179 (24.4%)	135 (18.4%)		
ACC/AHA lesion, B2/C type	590 (76.3%)	923 (72.7%)	0.08	565 (76.9%)	537 (73.1%)	0.10	
Chronic total occlusion lesion	126 (16.3%)	263 (20.7%)	0.02	124 (16.9%)	131 (17.8%)	0.68	
Bifurcation lesion	71 (9.2%)	126 (9.9%)	0.64	69 (9.4%)	66 (9.0%)	0.86	
In-stent restenosis lesion	58 (7.5%)	53 (4.2%)	<0.01	56 (7.6%)	35 (4.8%)	0.03	
Severely calcified lesion	199 (25.7%)	199 (15.7%)	<0.01	183 (24.9%)	121 (16.5%)	<0.01	
Multivessel PCI	130 (16.8%)	186 (14.6%)	0.21	121 (16.5%)	102 (13.9%)	0.19	
Treated lesions			0.15			0.23	
Left main coronary artery	15 (1.9%)	13 (1.0%)		13 (1.8%)	6 (0.8%)		
Left anterior descending artery	400 (51.7%)	689 (54.3%)		380 (51.7%)	390 (53.1%)		
Left circumflex artery	135 (17.5%)	237 (18.7%)		130 (17.7%)	145 (19.7%)		
Right coronary artery	223 (28.8%)	331 (26.1%)		212 (28.8%)	194 (26.4%)		
Intracoronary imaging use per patients	266 (34.4%)	505 (39.8%)	0.02	253 (34.4%)	278 (37.8%)	0.19	
Number of treated lesions per patient	1.18 ± 0.43	1.16 ± 0.40	0.16	1.18 ± 0.42	1.15 ± 0.40	0.23	
Number of stents per patient	1.37 ± 0.68	1.31 ± 0.62	0.07	1.37 ± 0.68	1.30 ± 0.62	0.07	
Mean stent diameter, mm	3.08 ± 0.43	3.14 ± 0.45	<0.01	3.08 ± 0.43	3.14 ± 0.45	0.01	
Total stent length, mm	34.84 ± 21.73	32.87 ± 21.43	<0.01	34.80 ± 21.61	32.89 ± 22.82	0.10	
Rotational atherectomy	1 (0.1%)	3 (0.2%)	0.99	1 (0.1%)	3 (0.4%)	0.62	
Procedural complications							
Slow flow or no-reflow	0 (0.0%)	7 (0.6%)	0.09	0 (0.0%)	6 (0.8%)	0.04	
Dissection	5 (0.6%)	7 (0.6%)	1.00	5 (0.7%)	5 (0.7%)	1.00	
Side branch occlusion	4 (0.5%)	5 (0.4%)	0.95	4 (0.5%)	1 (0.1%)	0.37	
Distal embolization	2 (0.3%)	5 (0.4%)	0.91	2 (0.3%)	3 (0.4%)	1.00	
Acute thrombosis	1 (0.1%)	2 (0.2%)	1.00	1 (0.1%)	1 (0.1%)	1.00	
Stent migration	1 (0.1%)	1 (0.1%)	1.00	1 (0.1%)	1 (0.1%)	1.00	
Coronary perforation	0 (0.0%)	0 (0.0%)	1.00	0 (0.0%)	0 (0.0%)	1.00	

Values are presented as numbers (percentages) or means ± standard deviation. NA, not applicable due to exclusion from propensity-score matching; SMD, standardized mean difference

ACC = American College of Cardiology; AHA = American Heart Association; DM = diabetes mellitus; MI = myocardial infarction; PCI = percutaneous coronary intervention.

### Incidence of Primary and Secondary Outcomes

**Fig 1:**
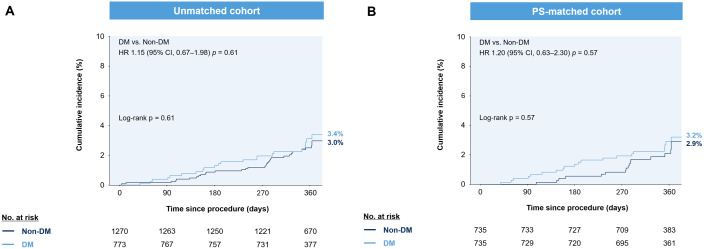
The occurrence of MACE in DM and non-DM patients. (A) Unmatched cohort, (B) PS-matched cohort CI = cumulative incidence; DM = diabetes mellitus; HR = hazard ratio; PS = propensity score.

At 12 months, the cumulative incidence of 12-month MACE was similar between the DM and non-DM groups, both before and after propensity score matching. In the unmatched cohort, the HR for MACE in the DM group was 1.15 (95% CI: 0.67–1.98; p = 0.61), and after matching, the HR was 1.20 (95% CI: 0.63–2.30; p = 0.57). (**Fig 1**) The rates of secondary outcomes, including all-cause death, cardiovascular death, myocardial infarction, and repeat revascularization, were also similar between the groups. (**Table 3**)

**Table 3:** Cumulative incidence of the primary and secondary outcomes at 12 months.

	Unmatched cohort				Propensity score-matched cohort			
	DM (n = 773)	Non-DM (n = 1270)	Hazard ratio	*P* value	DM (n = 735)	Non-DM (n = 735)	Hazard ratio	*P* value
**The primary outcome**								
MACE	22 (2.8%)	32 (2.5%)	1.15 [0.67–1.98]	0.61	20 (2.7%)	17 (2.3%)	1.20 [0.63–2.30]	0.57
**Secondary outcomes**								
Target-lesion failure	12 (1.6%)	18 (1.4%)	1.12 [0.54–2.32]	0.77	10 (1.4%)	12 (1.6%)	0.85 [0.37–1.97]	0.71
Death								
From any causes	16 (2.1%)	17 (1.3%)	1.57 [0.79–3.10]	0.19	16 (2.2%)	11 (1.5%)	1.47 [0.68–3.17]	0.32
From cardiac causes	3 (0.4%)	7 (0.6%)	0.72 [0.18–2.77]	0.63	3 (0.4%)	6 (0.8%)	0.51 [0.13–2.03]	0.33
Myocardial infarction	2 (0.3%)	3 (0.2%)	1.11 [0.19–6.64]	0.91	2 (0.3%)	1 (0.1%)	2.04 [0.18–22.5]	0.55
Repeat revascularization	19 (2.5%)	26 (2.0%)	1.23 [0.68–2.22]	0.50	17 (2.3%)	12 (1.6%)	1.45 [0.69–3.04]	0.32
TLR	7 (0.9%)	9 (0.7%)	1.30 [0.48–3.49]	0.60	5 (0.7%)	5 (0.7%)	1.02 [0.30–3.53]	0.97
TVR	11 (1.4%)	13 (1.0%)	1.42 [0.64–3.16]	0.39	9 (1.2%)	5 (0.7%)	1.84 [0.62–5.49]	0.27

DM = diabetes mellitus; MACE = major adverse cardiovascular events; TLR = target lesion revascularization; TVR = target vessel revascularization.

### Risk Factors for MACE

Univariable and multivariable analyses did not identify DM as a significant predictor of the primary outcome in the unmatched cohort. In the multivariable analysis, the HR for MACE in patients with DM was 1.07 (95% CI: 0.61–1.89; p = 0.82).

## Discussion

In this study, we evaluated the clinical safety and effectiveness of the Cre8 and Cre8 EVO stents in a real-world cohort of patients undergoing PCI, with a particular focus on those with DM. Our findings indicate that despite the higher prevalence of clinical and angiographic risk factors including CKD, multivessel disease, and severely calcified lesions in the DM group, the 12-month incidence of MACE was comparable between patients with and without DM. This suggests that the Cre8/Cre8 EVO stents may offer an effective treatment option in diabetic patients, addressing some of the unique challenges posed in this high-risk population. Furthermore, propensity score matching reinforced these results, showing no significant difference in MACE between the two groups, indicating the potential of the Cre8 and Cre8 Evo stents to achieve favorable outcomes in diabetic patients in a real-world clinical practice.

The advent of contemporary coronary DES has decreased the frequency of stent thrombosis and the necessity for recurrent revascularizations. Nevertheless, the presence of DM continues to pose a challenge, as patients with DM tend to have higher severity of coronary artery disease at the time of PCI, often followed by worse clinical or procedural outcomes thereafter. [27] DM is associated with proinflammatory conditions that enhance the vasculo-proliferative responses to stent-mediated arterial injuries, and disturbances that accelerate vascular atherosclerosis. Consequently, DM has consistently been a subgroup of interest for clinical trials that assess the efficacy of DES, and many studies have been designed specifically for patients with DM. Nevertheless, there is still a paucity of data on contemporary DES for patients with DM, and the optimal DES for these high-risk patients remains an unresolved issue. In a generation gifted with numerous different types of DES, the selection of the most suitable stent type is a multifaceted problem that ought to consider the stent platform, the polymer and the drug delivered, as well as clinical presentations or comorbidities.

The potential advantages of the Cre8 and the Cre8 Evo stents are 1) the absence of polymer coating, 2) thin stent struts and 3) the use of amphilimus, an amphiphilic carrier. Polymer coating in DES allows for the control of drug releases from the stent, ensuring slow and thorough local application of the antiproliferative drugs. However, polymer coating becomes functionless once the drug release is complete, and it may in fact affect the long-term patency of the DES. Polymers have been associated with local inflammatory reactions, the development of new atherosclerotic plaques and incomplete stent endothelialization, which may lead to late and very-late thrombotic events. [18,28] Diabetes is already a condition characterized by chronic vascular inflammation, potentially making patients with DM more susceptible to polymer-related inflammatory or hypersensitivity reactions. Such speculations were tested on various stent types with different polymer coatings, such as biodegradable polymers. Biodegradable polymers were considered as a midway solution between polymer and no polymer but failed to prove superior post-PCI outcomes in patients with diabetes. [29] These certain drawbacks of polymer coating led to the development of polymer-free stents.

In addition to the absence of a polymer coating, the abluminal reservoir technology unique to the Cre8 stents allows for a more uniform release of the drugs without the need for polymers. According to Fick’s law of diffusion, the release of drug over time is proportional to the drug concentration gradient and the area of contact. The abluminal reservoirs are dug directly into the stent surface using laser technology, which are then loaded with the drug formulations. The uniform distribution of the abluminal reservoirs on the stent surface allows for a uniform drug release. The drug release is also targeted specifically towards the vessel wall, with minimal or no release of the drug into the bloodstream, minimizing systemic effects. Drug release from macro-porous drug reservoirs is slower than that from a stent with direct coating of the drug and distinguishes the Cre8 stents from other polymer-free stents. The Cre8 stents endorse peak drug tissue concentration during the first days, 65 ~ 70% drug elution within 30 days and complete drug elution within 90 days. [12,28] The polymer-free Biofreedom™ stent reports a peak concentration of the drug released at 2 hours, and complete drug elution at 30 days. [30]

Another unique and important distinction of the Cre8 stents is the usage of Amphilimus™, a combination of Sirolimus and fatty acids. Diabetic cells show resistance to immunosuppressants used in DES via various mechanisms. For example, diabetes seems to directly hinder the effects of mammalian target of rapamycin (mTOR) inhibition on vascular smooth muscle cell (VSMC) proliferation, mandating at least a tenfold higher drug concentration in order to achieve a similar level of inhibition. [31] Diabetes and hyperinsulinemia are also associated with an increase in leptin levels, which contributes to in-stent restenosis by regulation of endothelial function and angiogenesis. [32,33] In patients with diabetes, higher concentrations of immunosuppressants are needed to block leptin-induced hyperplasia. [34] To overcome such disadvantages conferred by DM, very high drug concentrations are required – or a more efficient way of drug delivery. Amphilimus™ takes advantage of the fact that fatty acid utilization is elevated in diabetic myocardium, increasing uptake of the drug-fatty acid combination. Fatty acid molecules are also able to penetrate the lipid-laden cell membrane more easily, enabling improved drug delivery without higher concentrations. [12,35,36]

The results of our study are in line with previous results of comparison with various stent types. In the NEXT trial, Cre8 stent showed superior clinical outcomes compared with Taxus stent at 5-year follow-up. [16] Cre8 stents also showed better safety and efficacy compared with biolimus-eluting Nobori stent in patients with diabetes, while outcomes were similar for both stents in non-diabetic patients. [14] In RESERVOIR trial, neointimal volume reduction was higher in the Cre8 stent group compared with the everolimus-eluting Xience stent in a subgroup of poor glycemic control. [13] More recently, the SUGAR trial has demonstrated non-inferiority, and possible superiority, of Cre8 stents compared with the zotarolimus-eluting Resolute Onyx stent in patients with DM. [20] Overall, the Cre8 and Cre8 Evo stents demonstrate acceptable cardiovascular outcomes in patients with DM.

### Limitations

Our study has several limitations. First, being an observational cohort study, selection bias and unmeasured confounding factors cannot be completely excluded despite the well-balanced PS matching results. Second, being a prospective registry rather than a randomized trial, the study was not formally powered to test a pre-specified hypothesis. Thus, our results likely serve as a hypothesis-generating signal towards improved outcomes of PCI in diabetic patients rather than definitive evidence, and should be interpreted with caution. Third, the propensity-score matched cohort only had 735 patients per group, which meant that the sample size was too small to allow for meaningful subgroup comparisons such as insulin-treated versus orally treated diabetes. Future studies with larger cohorts are needed to explore this. Fourth, the follow-up period was relatively short; longer-term studies are needed to more strongly establish the benefits of Cre8 stents in the diabetic population. Notably, prior studies have shown that although the risk of stent thrombosis is highest during the first month after PCI, stent thrombosis can manifest beyond 12 months. [37] Nevertheless, it is worth noting that landmark analyses from more recent studies with newer-generation DES have consistently demonstrated that the majority of stent thrombosis occur within the first month after implantation, with gravest outcomes. [37,38] Further, to the best of our knowledge, this is the first real-world study which demonstrates comparable 1-year clinical outcomes of the Cre8 amphilimus polymer-free stent in patients with diabetes compared with patients without. Fifth, the occurrence of clinical outcomes appears to be relatively low compared with Western counterpart studies, which could have contributed to the lack of statistically significant difference in cumulative incidence of the outcomes between the diabetic and non-diabetic populations. However, the event rates for adverse cardiovascular outcomes after PCI have been relatively lower in studies based on East Asian populations, with similar event rates compared to this study. [39,40]

## Conclusions

These results underscore the potential for PF-AES to become a preferred option for PCI in patients with DM, prompting further investigation into their long-term benefits.

## Abbreviations

AES: Amphilimus-eluting stent
DES: Drug-eluting stent
DM: Diabetes mellitus
HR: Hazard ratio
MACE: Major adverse cardiovascular events
MI: Myocardial infarction
PCI: Percutaneous coronary intervention
PS: Propensity score
RCT: Randomized clinical trial
